# *Integrated physical education* and medicine in general physical education at universities in *the age of educational technologies*

**DOI:** 10.1186/s12909-023-04440-9

**Published:** 2023-06-22

**Authors:** Yunfei Niu

**Affiliations:** Physical Education Department, Tangshan Normal University, Tangshan, Hebei China

**Keywords:** Critical thinking, First aid, Integrated lesson, Interactive educational environment, Mobile applications, Physical education

## Abstract

**Background:**

The integration of training sessions into modern education is of vital importance for such disciplines as Physical Education and First Aid for the non-core specialities. This research explored the opportunities to introduce a pilot programme for Sports Medicine based on the First Aid and Fitness Tests applications to develop critical thinking skills in students using an indirect learning method.

**Methods:**

This research used the Fitness Tests application developed by the ConnectedPE software company. The software contains more than 30 fitness tests and indicates the goal, equipment, procedure and standards so that students can easily and accurately complete all tasks and improve their fitness. The experimental group involved 60 first-year students (25 females and 35 males). The average age is 18.2 years. The control group involved 28 males and 32 females with an average age of 18.3 years. Students were assigned randomly to groups to ensure the experiment’s validity.

**Results:**

The analysis of the pre-test and post-test of Critical Thinking Skills Success showed significant improvements in critical thinking skills (Z = -6.755 at p = 0.00) based on the integrated sports medicine programme. A negative correlation was observed between the post-test scores of Critical Thinking Skills Success and the Integrated Sports Medicine Test (r = -0.280, p < 0.05).

**Conclusions:**

This article fills a gap in research on the possibility of integrating physical education and medicine into one ICT-based university course that would optimise study hours and develop critical thinking. The research’s scientific value is to promote the discussion about the absence of a unified standard for the basic sports training of young individuals on a global scale. The practical significance lies in the enhanced development of critical thinking skills among students through integrated sports training sessions, as opposed to the conventional lecture format. The other important finding is the fact that the use of mobile applications and the development of a general programme in sports medicine have no positive impact or correlation with the academic outputs of students in these two disciplines. The research results can help educators to update curricula on physical education and extracurricular pre-medical training at universities. The perspective of this research is to integrate physical education with other academic disciplines, such as biology, mathematics, physics, and others, to determine the feasibility of this integration and investigate its effect on critical thinking.

## Introduction

Advanced technologies and changes in the education system increase the importance of physical education at universities and it is becoming essential. Modern education does not only imply educational activities. It also includes physical activity that contributes to the health and performance of students [1].

Nevertheless, with the evolution of the education system and the integration of novel technologies, there arises a continual need to update physical education methodologies to align them with contemporary standards. Currently, the integrated method of physical education is the most relevant since it includes not only exercises but also covers medical aspects [2, 3]. This article assumes that the integration of physical education and medicine lessons, along with modern technologies, will help students develop critical thinking skills and pay attention to both physical fitness and competence in the most common diseases. This is crucial since the study hours for non-core disciplines are limited and updated programmes should use the study hours to the maximum benefit. This article is an important contribution to the scientific field of physical education development. It offers new ideas and approaches to the integration of physical education and medicine in the education of university students. The study of first aid and physical education in one course is highly relevant for university students for several reasons. Firstly, knowledge of first aid can be critically important in case of emergencies, for example, injuries, fainting, heart attack, and so forth. Secondly, the study of physical education allows students to maintain their physical and mental health. The modern university lifestyle, often accompanied by sedentary work and stress, can lead to diseases associated with a lack of physical activity [4, 5]. In this context, a physical education course can help students develop a healthy lifestyle, strengthen their body, and reduce stress levels.

Finally, integrated lessons combining physical education and medical techniques can deepen students’ understanding of the relationship between physical activity and health. This knowledge is important for the prevention of chronic diseases. The scientific novelty lies in the use of ICT for the most successful integration of physical education and a practical medicine course.

### Literature review

Integrated learning has emerged as a global trend in advanced education since the mid-20th century, with Europe and Asia adopting an integrated approach in curriculum, textbook design, and teaching practices across various educational levels [6]. Simultaneously, physical education has aimed to enhance physical fitness among students and improve their overall health. The introduction of innovative teaching methods emphasizes the exchange between teachers and students, fostering greater student interest in sports and transforming their perspectives on health-related issues. This aligns with the contemporary philosophy of physical education development worldwide. At the onset of the 21st century, Chinese students’ physical health and overall fitness have been on a steady decline. For over a decade, indicators such as lung capacity, speed, strength, and endurance have exhibited worsening trends [7]. Traditional physical education primarily focuses on the learning process and acquisition of fundamental knowledge, often neglecting the active participation of students. Consequently, a gap has emerged between teaching and learning within the field [8]. In recent years, Chinese colleges, universities, and educational institutions worldwide have begun integrating comprehensive physical education programs that combine theoretical and practical components, incorporating various curricula, training exercises, and physical skill development [7].

Advancements in the digital age have facilitated the utilization of gadgets not only for entertainment and information exchange but also for collecting health-related data and proposing optimal approaches to physical training. The availability of effective feedback mechanisms enables students to engage in regular physical education activities. By comprehending the value and focus of the proposed training programs, students are more motivated to participate. University physical education teachers can leverage this data to design tailored physical training exercises that improve the quality of education and enhance student motivation [9].

Integrated education programmes are positioned as an alternative educational practice, developed based on two or more science areas to achieve the academic goal in each discipline. However, sceptics underline that the integration practices in education lead to a knowledge decline among the students because there is no full involvement of students in the discipline. The pedagogical impact mentioned above is primarily manifested in the increased level of student engagement and positive attitudes towards education. Furthermore, it facilitates meaningful interdisciplinary communication, which contributes to a dual effect [10]. At the level of school education, it is proposed to integrate the personal diary into physical education. The strategy will help students become more mindful of their health, as well as learn how to describe sports experiences concisely and succinctly to meet educational goals such as self-knowledge and self-improvement [11].

Physical education can positively influence the development of critical thinking skills based on the inductive thinking model because the traditional stimulus-response learning mode is considered less effective than the mediation model [12]. Using the integrated sports medicine programme implies that students should not only memorize facts from textbooks or repeat exercises but focus more on vaguely formulated tasks. This undertaking can be likened to problem-solving situations that necessitate both coordinated teamwork and swift response times [13].

The integration of physical education and medical science into the complete discipline is currently found in school rather than university education. The scholars discuss the first aid programme recommended for elementary schools. The programme involves medical skills and physical education, covering fitness, general motor skills, sense of rhythm, dance, gymnastics, and outdoor games [14]. One of the approaches to improve the quality of physical education is the use of digital technologies such as the iPad, camcorders, mobile applications and wearable gadgets (smart watches) related to health. These advancements open up novel opportunities for enhancing physical education through the utilization of video analysis techniques, as well as the exploration of anatomical and physiological responses.

Digital technology can be used as a scoreboard and a specialised platform to provide students with interactive classroom management using a timer, music display and microphone. At the same time, it was found that gadgets (laptops, tablets, mobile phones and other applications used in physical education) can improve the students’ motivation, develop motor skills, increase inclusion and improve group belonging and cohesion [15].

The use of mobile applications and ICT in the physical education of students at universities is becoming increasingly popular all over the world. Mobile applications are actively used tools to track students’ physical activity and create personalized training programs [16, 17]. The latter may include various types of exercise, as well as dietary and rest recommendations. Thus, for instance, such applications as MyFitnessPal, Nike Training Club, and others provide students with access to statistics and reports. This information allows users to track their progress and motivates them to further improve their results. ICT also serves as an instrument to create virtual training programs and simulators. These projects help students learn various types of physical exercises and techniques for performing them without the risk of injury [18].

In general, universities worldwide tend to integrate mobile applications and ICT into physical education. This trend continues to develop [19]. Nevertheless, the degree of integration may vary depending on the university and region, as well as on the availability of the necessary resources and technologies.

### Problem statement

The analysis of existing literature indicates a limited adoption of the proposed progressive method, known as learning integration, in higher education settings, both within China and internationally. Considering that life safety and physical education are two basic issues that each needs to maintain a normal life, students need to learn and test the effectiveness of anatomy, medical science, and sports training. The general educational programme can be an excellent alternative to the traditional one. It can increase the academic motivation of students who learn a certain speciality and pay relatively less attention to general disciplines. These educational viewpoints substantiate the fact that a school curriculum focusing on the Fundamentals of life safety may not offer a comprehensive framework for comprehending the intricacies of human anatomy and acquiring the necessary skills to sustain a healthy lifestyle. Therefore, this knowledge is important for everyday practices, which will increase the interest of students and join the development of physical skills and the expertise to provide first aid, identify the type of damage and learn more about the internal human organs.

The research purpose is to develop a pilot digital programme known *as Sports Medicine* that involves sports training and basic knowledge and skills improvement in health literacy among legal-age individuals in China. To achieve this goal, the scholars perform the following tasks. At first, they developed an integrated training programme for second-year students, using elements of physical education programmes and first aid for injured individuals. Furthermore, the scholars assessed the efficacy of the program by conducting a comparative analysis of the fitness levels between students who completed an integrated sports medicine program and those who underwent conventional physical education and first aid training. Third, the scientists collected new empirical data on the impact of integrated lessons on the critical thinking skills of sophomores. The proposed testing approach will help to evaluate the positive effect of integrating physical education and knowledge about first aid into complete discipline, as well as expand the discussion on critical thinking skills among students.

## Methods and materials

### Research tools

**Fig. 1:**
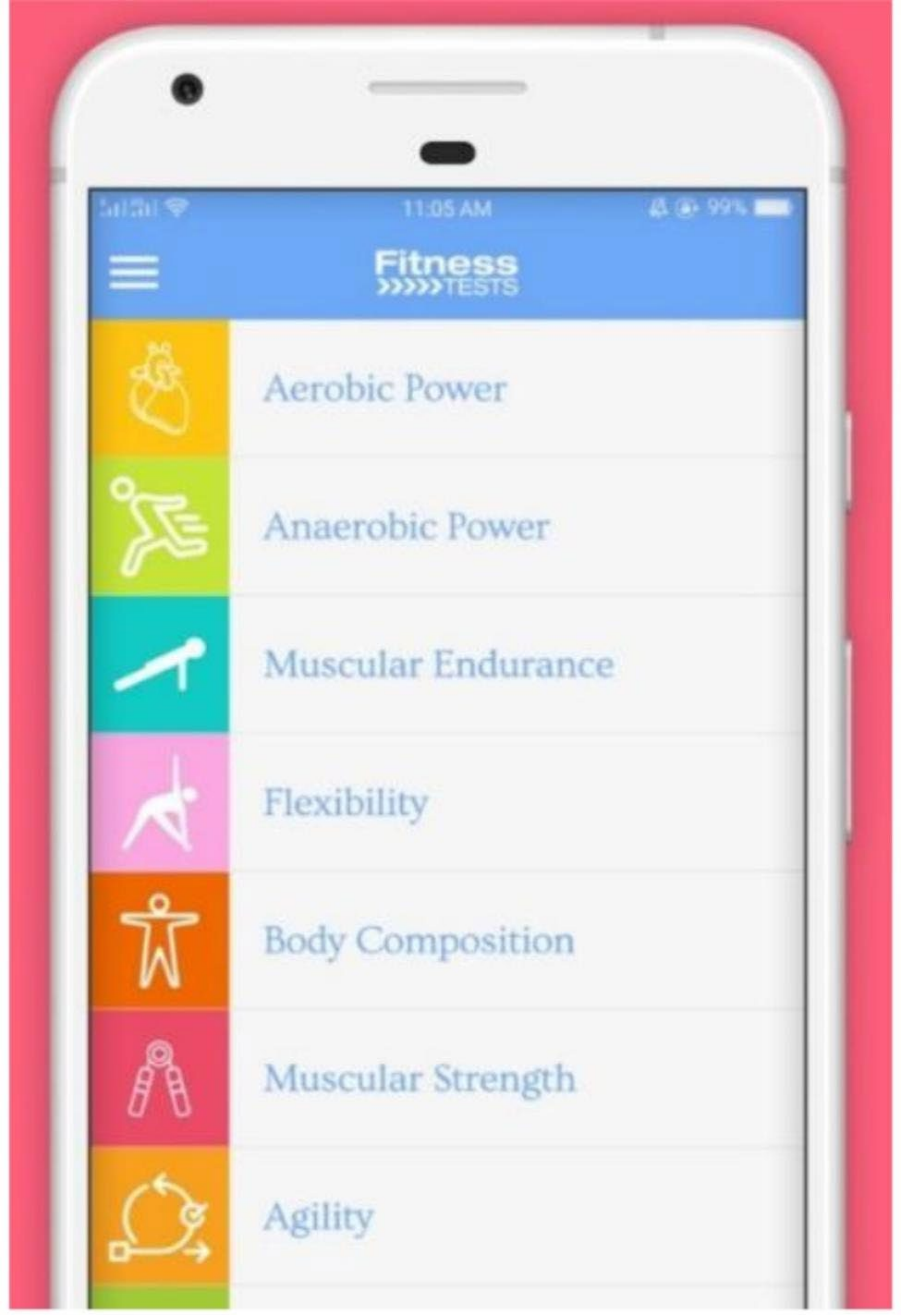
Fitness Tests application interface

This research used the *Fitness Tests* application developed by ConnectedPE software company. The software contains more than 30 fitness tests and indicates the goal, equipment, procedure and standards so that students can easily and accurately complete all tasks and improve their fitness. The application interface is illustrated in Fig. 1.

The rationale for choosing the application is its content. It included tests that can be easily integrated into the system of teaching physical education at the university. This implies a certain standard of preparation. It was also important that this application offered a variety of physical activities, including certain equipment that can interest students and increase their engagement.

**Fig. 2:**
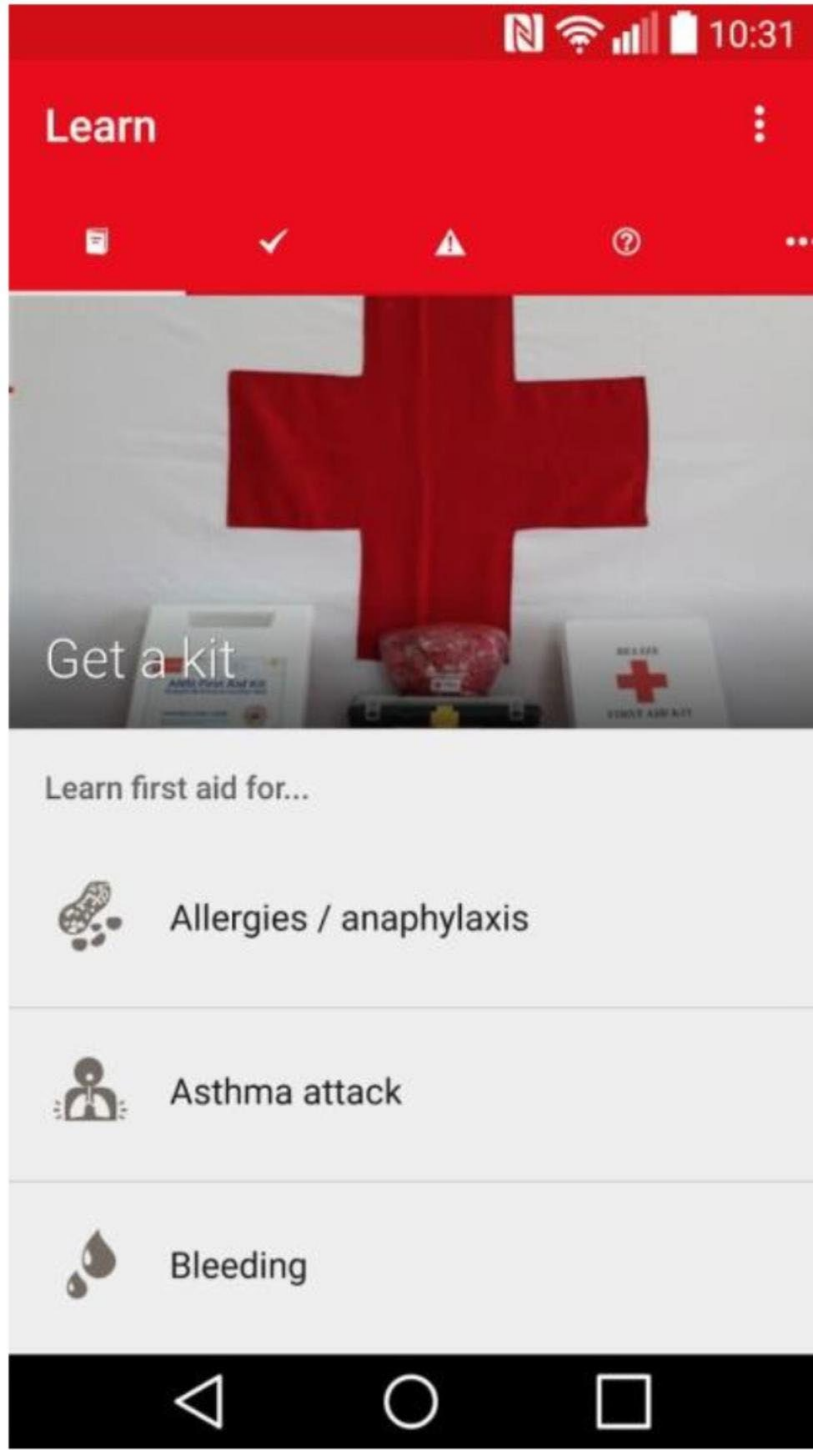
First Aid application interface

For integrated training, the First Aid application from the IFR company was used. This software ensures instant access to the information needed to provide first aid in the most common cases. They involve heart attack, stress, poisoning, bone fractures, allergies, asthma, bleeding, and so on. There are videos, interactive quizzes and step-by-step instructions to provide first aid for injured individuals. The application is available in Chinese and other languages in Simplified and Traditional formats. The interface is illustrated in Fig. 2.

This application was part of the study due to its unique features that other apps did not have. The app provides the information in an interactive form, involving pictures, videos, and so forth. In this case, it is not the format of a book, that requires reading to obtain any information. In addition, the presence of the Chinese language was essential to overcome the language barrier. Knowledge of first aid was among the most important in terms of saving life and having basic knowledge to maintain the health of students and the people around them.

The choice of this technique was due to the presence of two forms: pre-testing and post-testing. This feature was remarkable among the other methods used for this study. The “Limitations” section indicates that this technique has several methodological limitations to consider when interpreting the findings. However, the respondents studied under the same methodology before and after the experiment. Therefore, it was possible to see whether the impact was effective or ineffective. In addition, this technique allowed the authors to compare the results quantitatively.

### Research measurements

To assess the impact of integrated sports medicine on students’ critical thinking skills, the *Critical Thinking Skills Success* methodology developed by the American researcher L. Starkey was applied. The advantage of the methodology is that the test is paired, which means that it ensures pre-test and post-test data collection [20]. Both forms consist of 30 questions with four possible answers, one of which is correct. One correct answer is 1 point. The technique is single-factor and is intended for individuals over 16 years [21]. This will help to test the initial findings and analyse the impact of integrated activities on human critical thinking skills [12, 22]. The validity of the test was verified with Cronbach’s Alpha (α = 0.887).

Based on the sports medicine programme, *the Integrated Sports Medicine Test* was developed. The first part was based on parameters such as body mass index, lung capacity, running 50 m, forward tilt of the torso from a sitting position, long jump from a place, pulling up in 1 min (males) and lifting the torso from a lying position to a sitting position (females), as well as running 1000 m for males and 800 m for females [23–25]. For physical exercises, each participant could receive up to 50 points.

The second part of the exam involved filling in a form based on the materials learnt in the first aid programme. These were 50 questions with four possible answers, one of which was correct. The proposed questions covered the first aid issues such as asthma, burns, bleeding, broken bones, asphyxiation, head trauma, absence of respiration, and unconsciousness. For this module, each participant could also get up to 50 points. The maximum score for the post-test is 100 points. The successful performance of the experimental group required additional equipment, including a metronome, a stopwatch, sound prompting signals, chalk, a tape measure, a dynamometer, marking cones, a tennis ball, and a measuring tape. It was also necessary to have a flat non-slip surface, treadmills with marked distances in meters, a set of horizontal bars for pull-ups and a solid wall. These resources and facilities were located at *the Faculty of Physical Education and Gymnasium at Tangshan Normal University* However, a multimedia whiteboard and audio resources were added to make the most of the applications, including entering the personal scores of participants into a spreadsheet from the application. The control group required less equipment and simple conditions for training. Balls for contact games, a stopwatch and roulette were used. The traditional training was mainly based on the collective repetition of movements. This refers to the usual running, physical exercises, and sports games.

During the training in First Aid application, no special materials were required. The application was broadcast on the screen in the gym, and students could make some notes on their phones and ask questions. The information provided by the application was the core on which additional information, comments and questions from the audience were stored.

### Participants

The experimental group involved 60 first-year students (25 females and 35 males). The average age is 18.2 years. The control group involved 28 males and 32 females with an average age of 18.3 years. Students were assigned randomly to groups to ensure the experiment’s validity. The random number method was used and met the student’s number in the educational journal. Parameters such as height, weight and lung capacity were also taken into account with the permission and consent of the respondents.

### Research design

In the first stage, a pre-test was conducted using the Critical Thinking Skills Success method both in the control and experimental groups. Regular paper forms were used. After the test, a focused pedagogical framework was introduced to students. A member of the experimental group attended an hour and a half of the integrated sports medicine programme once a week. Each of the 21 sessions began with warm-ups to avoid injury and warm muscles.

During the training in the gym, *Fitness Tests* were published, including instructions, videos, and music as needed. This experimental programme included the following types of physical activity from the application: 20 m Shuttle Run, 12 min Run, 1.6 km Run, Harvard Step Test, Queens Step Test, 300/400/800m Run Tests, RAST tests, Handgrip Dynamometer, 1RM Bench Press, 1RM Leg Press, Timed Push-ups, Pull Up Test, Squats Test, Sit and Reach Test, Trunk Rotation Test, Shoulder Elevation Test, Waist Circumference, Body Mass Index, Waist-Hip Ratio, Vertical Jump Test, Standing Broad Jump, 35 m Sprint, 50 m Sprint, Illinois Agility Test, T-Test, Stork Balance Test, Standing Balance Test, Alternate Hand Wall Test, Soft Drink Can Test, Ruler Drop Test and others that students from the experimental group could try for free outside the traditional physical education. These activities involve different types of training selected by the physical training teacher. All results were entered into the scoreboard at the end of each lesson. One hour was allotted for physical education according to this programme. Following the initial 30-minute interval, the students proceeded to engage with the First Aid application, wherein the topics were sequentially revisited. After the second half hour, intended for the development of pre-medical skills, the students returned to physical training. As part of this programme, the experimental group covered all the topics available on the application, from allergies to the resuscitation of a lifeless patient. A total of 21 topics were learnt.

Comparing the effectiveness of the traditional and integrative interactive programmes, at the final stage, members of different groups had to meet the provided standards following the current CNSPFS approved by the Chinese government in 2014 [25]. It stands for “Chinese National Student Physical Fitness Standard”. This state standard is for schoolchildren. There is no generally accepted university standard developed yet. However, CNSPFS can be used for this research because it involves the analysis of the freshmen’ behaviour patterns regarding physical training.

The control group exercised in the gym for one hour, once a week, and then had a one-hour lesson in First Aid. No interactive tools were used. The physical education lesson was held in the traditional format and included a warm-up, contact games (football, basketball, and volleyball) and traditional standards and activities such as shuttle running, long jumps, push-ups, ball throwing and more. The first aid lesson in this group lasted one hour and was based only on textbook materials, which also included illustrations of instructions, similar to the First Aid application. In the control group, the acquisition of first aid knowledge took place through classroom-based instruction, predominantly delivered via teacher-led lectures. The topics were the same as those proposed to the experimental group in the application to create a playing field.

After six months, a Critical Thinking Skills Success post-test was conducted. The scholars collected responses within three training days using paper forms. After that, participants in the experimental and control groups took a physical test based on the CNSPFS standard in China and also filled out a form with questions on first aid, which was part of the Integrated Sports Medicine Test. Moreover, the scholars gathered, synthesized, and analyzed the collected results.

### Data analysis

Data processing was carried out using the SPSS Statistics 23 programme. Spearman’s rank correlation coefficient was used to analyse the correlation between two variables. To compare the outcomes among different groups, a Nonparametric Mann-Whitney U test for two independent samples was utilized. Additionally, the Wilcoxon signed-rank test was employed to evaluate the changes between the pre-test and post-test within the respective critical thinking samples.

### Ethical issues

The present research followed high ethical standards because each participant in the control and experimental groups signed the informed consent and was informed about the research goals. This study did not cause any physical or mental harm but was conducted for scientific purposes. Tangshan Normal University bore all financial costs related to the experiment. The scholar did not have any personal or financial benefit. The software development companies have not invested in this scientific research and were not considered interested parties. Some of the ethical limitations were that freshmen did not have the opportunity to choose between interactive and traditional educational environments. The experiment should be valid and trustworthy. The scholars admit the legal rights of the respondents and the successful implementation of the principle of utility.

### Limitations

The six-month experiment could potentially reduce academic motivation and involvement in the critical thinking post-test. The relatively small number of participants in the groups limits the research reliability. The frequency of lessons conducted once a week was determined by the curriculum of Tangshan Normal University, which could create preconditions for fatigue in the experimental group, which simultaneously learnt two disciplines.

The CNSPFS standard is not intended for students but for schools up to grade 12. At the same time, none of the respondents received auxiliary points as encouragement [25]. The absence of a standardized framework for general physical training in universities prompted scholars to conduct research specifically focusing on first-year students. Furthermore, they have outlined plans for future scientific discourse on related subjects to address this knowledge gap. The relatively high number of group participants (30 individuals in real-life conditions) can be considered a limiting factor, which increased the workload on teachers and left little for an individual approach to teaching physical education.

## Results

**Fig. 3:**
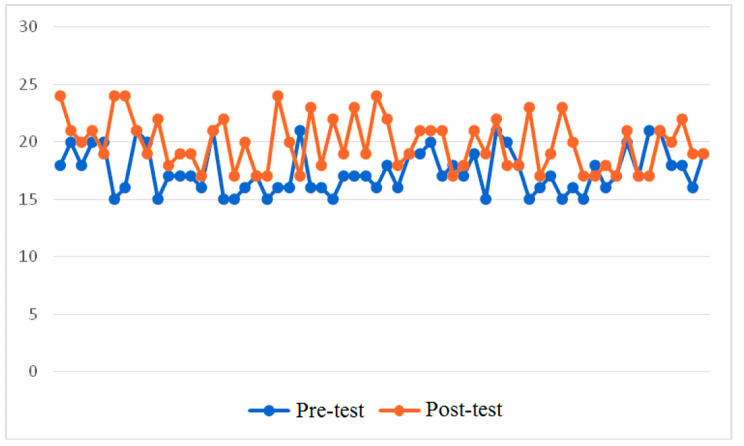
Critical Thinking Skills Success test before and after the training (Control Group)

Data analysis involved a comparison of pre-test and post-test results using the Critical Thinking Skills Success methodology to assess the effectiveness of the integrated lesson introduced for the development of critical thinking skills in students. The data for the control group is available in Fig. 3. The horizontal axis shows the sum of the points scored by the respondents, and the vertical axis separates the test results before and after the intervention.

In this group, the mean value for the pre-test was 17.47 points. The scale showed that the minimum number was 15 points, and the maximum number was 21 points. The standard deviation in this group did not exceed 1.944 points. The post-test results were high on average. The average score increased to 19.88 points and reached a maximum value of 24 points.

**Fig. 4:**
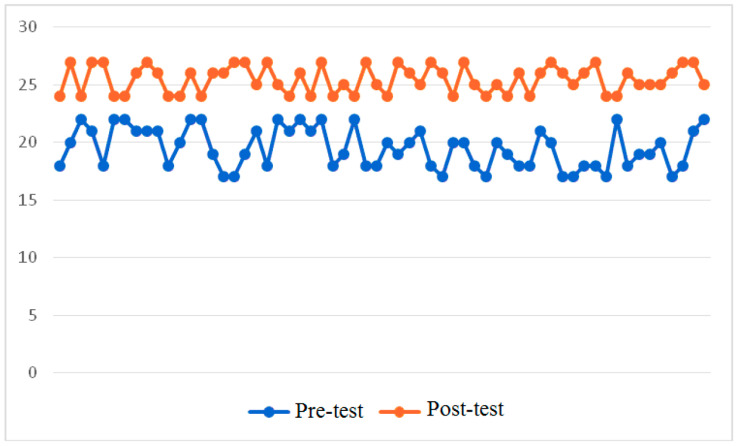
Critical Thinking Skills Success test before and after the training (Experimental Group)

Based on the schedule, traditional physical education and the first aid programme introduced new approaches to developing critical thinking skills. The Wilcoxon signed-rank analysis showed that in 44 cases there was a positive trend, in 7 cases, it was negative, and in 9 no changes in the results were identified. In this case, Z = -5.128, and p = 0.000. The anticipated impact demonstrated a favourable influence on the cultivation of students’ critical thinking abilities. However, situational factors may also influence the outcomes, for example, the prior experience from the pre-test, the involvement of students filling out the form, and others. In the next stage, the scholars investigated the pre-test and post-test results based on the Critical Thinking Skills Success method in the experimental group. The data is available in Fig. 4.

Figure 4 depicts that the interactive educational environment positively impacts the advancement of critical thinking skills in all 60 cases, resulting in notable enhancements in soft skills. In this group, the pre-test average was 19.52 points, and after the training programme implementation, it reached 25.47 points. The minimum score on the post-test was 24 points, although the lowest score was initially 17 points. The standard deviation was slightly higher in the pre-test, namely 1.742 points versus 1.186 points in the post-test.

The implementation of an integrated sports medicine lesson yields a beneficial impact on the enhancement of critical thinking skills in students. The research found that there were some differences between the groups according to the analysis of the Wilcoxon T-test: Z = -6.755 at p = 0.000. Based on their findings, the scholars concluded that the utilization of the integrated lesson as a pedagogical tool led to notable enhancements in critical thinking skills, surpassing the outcomes observed with traditional learning approaches solely reliant on educational materials.

In the second stage of the statistical analysis, the results of the Integrated Sports Medicine Test were compared. They involved practical assignments and theoretical knowledge of first aid. Data on average values are available in Table 1.

**Table 1:** Average values of the Integrated Sports Medicine Test depending on the intervention

ISMT						
*Groups*	*Average value*	*N*	*Standard deviation*	*Median*	*Minimum*	*Maximum*
Control group	77.80	60	7.073	77.00	64	90
Experimental group	72.07	60	6.045	73.50	62	81

According to the primary data analysis, the control group showed a higher result in physical training based on the traditional pedagogical approach. After the programme, the average value for the test was 77.80 points. In the experimental group, the recorded value was marginally lower at 72.07 points. Conversely, the control group exhibited a higher median, with scores of 77 points compared to 73.50 points in the experimental group. According to the minimum and maximum values, the control group showed better results than the experimental group. If in the control group, the minimum value was 64 points, then in the experimental group it was 62 points. The highest number of points in the experimental group was 81 points, and in the control group, it was 90 points. The results suggest that the traditional approach to physical education and first aid is no less effective than an interactive educational environment in an integrated lesson. In the experimental group, the learning process took at least double the amount of time to master medicine. The research delineated the specified approach and time constraints. The results comparison was the next step in the data analysis. The results are available in Table 2.

**Table 2:** Statistical analysis of the difference between the group values according to the Integrated Sports Medicine Test

	ISMT
*Mann–Whitney U test*	247.500
*Wilcoxon signed-rank test*	712.500
Z	-2.998
*Asymptotic significance (2-sided)*	**0.003**

The secondary statistical analysis showed a relatively significant difference at the level of p = 0.03 with acceptable p ≤ 0.05 between the groups with different pedagogical impacts trained within six months. The Mann-Whitney U test was 247.500. The traditional and expected type of influence was more effective than the experimental one. The scholars suggest that these results are based on the need to disperse attention between physical training and first aid knowledge. The scholars suppose that future research is needed to experiment with increased academic hours for medical training in the experimental group. However, the integrated sports medicine approach to education will not require a reduction in teaching hours, and this is one of the main reasons for the integration of core disciplines at the university. The experimental group showed poor results because the use of applications in physical education lessons was less desirable than personal communication in the traditional classroom. Both groups showed a relatively high score, exceeding 70 points.

Both teaching strategies can be used at the university. The potential improvements of each strategy are elaborated. The research investigates the relationship between critical thinking and academic achievements in sports medicine. The data is available in Table 3.

**Table 3:** Relationship between Critical Thinking Skills Success and Integrated Sports Medicine Test post-test scores

			ISMT	Post-test CTSS
*Spearman’s Rank correlation coefficient*	ISMT	Correlation coefficient	1.000	**− 0.280** ^*^
		Values (2-sided)	.	0.030
		N	120	60
	Post-test CTSS	Correlation coefficient	**− 0.280** ^*^	1.000
		Values (2-sided)	0.030	.
		N	120	60

Note: * – the correlation is significant at 0.05 (two-sided)

The analysis revealed a significant negative correlation (r = − 0.280) at the 0.05 level between the critical thinking and sports medicine tests. The results suggest that there is a significant negative relationship between critical thinking and physical fitness in the increase-decrease / decrease-increase relationships. Therefore, critical thinking as a soft skill is significant for high-quality physical training and mastering the key skills in first aid. The proposed approach underlines the importance of developing critical thinking at the university and confirms the importance of body-mind interaction.

## Discussion

The video graphics model is a common audiovisual media strategy for successful integration into physical education. Students can watch the physical training exercises, and track individual progress and needs, which influence productivity and help to correct mistakes immediately. The learning model based on augmented reality (AR) technology on a sample of schoolchildren is more effective than learning with the help of video resources [26]. The utilization of mobile technology as an instructional tool in physical education has the potential to enhance academic performance and foster heightened student motivation. It helps children learn and practice motor skills at their own pace. The most accessible and effective new technology in physical education is the photographic equipment so that students can understand their mistakes when performing exercises such as throwing a ball, long jumps, shuttle running and others. A system for monitoring, transmitting and processing information about sports performance based on feedback has also been developed. It provided students (trainers) with mobile devices and wireless sensors [17]. The scholars identified the main functions of the digitalisation of physical education.

Smartphone applications can be used as the main means of communication (scoreboard, interactive whiteboard, and display platforms) and classroom management tools (timer, music display, and microphones). Furthermore, these technologies possess the capacity to provide additional information such as feedback, lesson plans, and assessments while also enabling personalized adjustments by the unique needs and abilities of individual students. This facilitates the development of customized training programs [27]. A sample of first-grade children showed the effectiveness of the integrative teaching method for mathematics. Physical training helps to improve the quality of education compared to the traditional teaching of mathematics and physical education [28].

The approach discussed above differs from the results of this research. Although, the data were collected from a sample of a completely different age, which complicated the possibility to use the bridging principles. The effectiveness of the First Aid application was tested using samples of literate and illiterate individuals. The results showed that hand-drawn sketches with first aid activities were better understood by illiterate individuals compared to image-based interfaces. However, there was no clear difference in usage between literate and illiterate individuals when they used freehand drawing-based interfaces [29].

The simulation of the user interface for first aid was based on the user-centred design (UCD) recommended for schoolchildren from 7 to 12 years. The research proposes to use the AidHub application, based on a video game [30, 31]. However, the effectiveness of these integrative teaching methods has not been compared with the traditional ones and the effectiveness is still questioned. The physical training based on 20-minute episodes outside of the PE programme improved the academic achievements in walking and running. There is evidence that physical activity and academic performance are closely connected [2]. However, these data cannot be considered valid in this research.

The scientific literature proves that STEM education as an example of a transdisciplinary approach in pedagogy promotes the development of critical thinking skills among students, which coincides with the present experiment [32]. The CinQASE model (this acronym stands for Critical Thinking for Active and Collaborative Learning through Questioning, Analysis, Synthesis, and Evaluation ) has been developed and integrated into a school programme in physics. The learning modules such as problem-centred approach, individual work, collaborative critical thinking, teamwork, discussion, assessment and feedback have been proposed to students [33]. The perspective of using virtual feedback for physical training, skill acquisition by young gymnasts, self-esteem, as well as motivation in learning was investigated.

The results showed that the use of a simplified video-based learning tool with self-assessment tasks improved motor skills, as well as improved the motivation and self-esteem of young gymnasts in a short period [34]. The research confirms that the data collected on the integrated lesson as a form of learning has a positive effect on the critical thinking skills of an individual [12, 22]. However, the main difficulty in comparing the results is the lack of any empirical data on student samples. It proved the fact that education paid insufficient attention to physical education in universities around the world.

The utilization of digital technologies in physical education contributes to the development of critical thinking skills among students. They can analyze their training sessions, identify errors, and make informed decisions to enhance their skills. Moreover, digital tools enable them to collaborate, exchange ideas and arguments, fostering critical thinking through collaborative learning. The use of digital assessment and feedback mechanisms assists students in analyzing their performance and devising strategies to improve their achievements in physical activities [35]. Within an interactive educational milieu, learners have the opportunity to engage in collaborative endeavours, share insights, and scrutinize their training sessions. This enables the development of critical thinking skills through interaction and information exchange with their peers [36]. Thus, the active utilization of digital technologies in physical education strongly supports the development of critical thinking among students by stimulating analytical thinking, self-assessment, informed decision-making, and collaborative learning.

## Conclusion

The theoretical analysis showed that the main advantage of integrated lessons was the activation of different brain areas, as well as saving time, which was especially valuable for many teachers. In modern China, as well as throughout the world, no standards of physical education at universities exist, except for narrow-profile institutions that train professional athletes, police officers, and other professionals. To update university physical education programmes, integrated sports medicine based on digital technologies was developed and introduced into the educational process.

The research uses mobile applications such as Fitness Tests and First Aid. The research hypothesised that the use of an interactive educational environment would yield greater efficacy in facilitating sports training and enhancing the acquisition of first aid skills. However, the Integrated Sports Medicine Test showed that the traditional type of pedagogical intervention was more effective than the interactive interdisciplinary method. This means that the possibility of introducing digital technologies into university physical education is not as clear as the integration of physical education and medicine into a complete programme. The research revealed that both types of interventions ensured high results on the Integrated Sports Medicine Test, described as above average in both groups. The scholars found that an integrated lesson as a form of educational activity helped to improve the critical thinking skills of students.

The research findings empirically demonstrated that the interactive integrated learning platform for sports and medicine resulted in a consistent enhancement of critical thinking skills, as evidenced by the increase observed in participants’ performance on the post-test compared to their pre-test scores at the beginning of the experiment. Although the traditional model positively affected students’ academic achievements, it did not produce explicit or specific results. The research also revealed a significant inverse correlation (r = -0.280) between the results of the post-test Critical Thinking Skills Success and the Integrated Sports Medicine Test. This means that more attention should be paid to the physical and medical preparation of young individuals who entered high educational institutions, because brain activity is inextricably linked with the human body, and first aid skills can help to save a human life.

Interdisciplinary integration and digital technologies are more common in schools rather than in university education. The lack of similar empirical research encourages the scholars to promote the discussion about the results, as well as pave the way for other scientists to update physical education curricula at the university. Integrated education as a form of organisation is less common in high education because this system is more conservative and rigid. Although interactive lessons can be implemented in different educational environments to promote the interest of students in physical education, learning the first aid standards, and developing critical thinking skills in young individuals.

## Data Availability

The datasets used and(or) analysed during the current study are available from the corresponding author upon reasonable request.
